# Customizing Robot-Assisted Passive Neurorehabilitation Exercise Based on Teaching Training Mechanism

**DOI:** 10.1155/2021/9972560

**Published:** 2021-05-31

**Authors:** Yingnan Lin, Qingming Qu, Yifang Lin, Jieying He, Qi Zhang, Chuankai Wang, Zewu Jiang, Fengxian Guo, Jie Jia

**Affiliations:** ^1^Department of Rehabilitation Medicine, Huashan Hospital, Fudan University, China; ^2^Shanghai Electric GeniKIT Medical Science and Technology Co., Ltd., Shanghai, China; ^3^National Clinical Research Center for Aging and Medicine, Huashan Hospital, Fudan University, China

## Abstract

Passive movement is an important mean of rehabilitation for stroke survivors in the early stage or with greater paralysis. The upper extremity robot is required to assist therapists with passive movement during clinical rehabilitation, while customizing is one of the crucial issues for robot-assisted upper extremity training, which fits the patient-centeredness. Robot-assisted teaching training could address the need well. However, the existing control strategies of teaching training are usually commanded by position merely, having trouble to achieve the efficacy of treatment by therapists. And deficiency of flexibility and compliance comes to the training trajectory. This research presents a novel motion control strategy for customized robot-assisted passive neurorehabilitation. The teaching training mechanism is developed to coordinate the movement of the shoulder and elbow, ensuring the training trajectory correspondence with human kinematics. Furthermore, the motion trajectory is adjusted by arm strength to realize dexterity and flexibility. Meanwhile, the torque sensor employed in the human-robot interactive system identifies movement intention of human. The goal-directed games and feedbacks promote the motor positivity of stroke survivors. In addition, functional experiments and clinical experiments are investigated with a healthy adult and five recruited stroke survivors, respectively. The experimental results present that the suggested control strategy not only serves with safety training but also presents rehabilitation efficacy.

## 1. Introduction

New advances in technology and an increased upper extremity motor dysfunction lead to widespread adoption of robots in clinical rehabilitation poststroke. Robotic devices for upper extremity rehabilitation have the potential to deliver highly repetitive, task oriented, intensive, and quantifiable neurorehabilitation [1], which is perceived to be one of the most effective approaches for function restoration of the upper extremity. A 2018 Cochrane review [2] declares that robot-assisted therapy might improve activities of daily living, arm function, and arm muscle strength. Nonetheless, the effects depend upon the type of device, intensity, duration, amount of training, treatment program, and participant's residual functional ability. Aspects described above interest us to explore further in robotic devices for upper extremity rehabilitation.

The rapid development of many robotic devices has been seen during the past two decades. And the devices fall into two main classes: robots developed to compensate for lost skills (assisted devices) and robots designed to recover lost function by training (therapy devices) [3]. The main goal of the therapy devices is to assist therapists by customizing rehabilitation with high intensity, which is one of principles of stroke rehabilitation [4], while the therapy devices are categorized as end-effector-based robots and exoskeleton-type robots from a mechanical structure point of view. Meanwhile, passive, active-assisted, and active-resisted movement modes can be implemented in therapy devices and even bimanual training mode [5]. Passive movement activates the sensorimotor system through conveying proprioceptive information not only to sensory but also to motor cortices which has been well-documented [6–8]. And evidence suggests that passive movement is successfully applied in motor rehabilitation [9, 10]. The brain networks subserving passive movement in previous studies were shown in accordance with these ideas [11, 12]. Hence, rehabilitation providing passive movement is the crucial part to restoration of stroke survivors at the early stage or with greater impairment, especially for the upper extremity. Meanwhile, upper extremity robot offering passive movement is particularly urgent. However, the passive movement with motor incoordination between the shoulder and elbow appears in the upper extremity robot currently. And the movement trajectory is intermittent and deviates from the physiological activity of human due to the application of general industrial methods. Furthermore, the motor is controlled by the position merely and the upper extremity of patient follows the robot arm strictly, which contribute to the inflexibility. The robot is simply mechanical repetition. And the effect of passive movement by therapists is not achieved.

The aim of designs is not to realize a brand novel robot for stroke survivors but to fill an existing gap that customizes robot-assisted passive movement. Intending to make the passive movement patterns customized on the residual functional ability and embedded in therapist's track, the paper analyzes the design of the upper extremity robot with human-robot system and teaching training mechanism. Besides, functional and clinical experiments were conducted to verify the effectiveness and efficacy of proposed teaching training mechanisms.

Thus, the research presents a novel motion control strategy for robot-assisted passive neurorehabilitation exercise. The teaching training is developed to assist therapists with customized smoothing passive movement, based on the judgment of residual function and desired training trajectory by therapists.

## 2. Upper Extremity Robot Design

**Figure 1:**
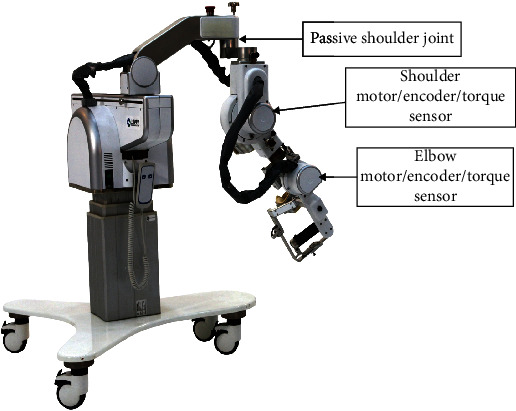
Mechanical structure of robot.

FELXO-Arm1 system manufactured by Shanghai Electric GeniKIT Medical Science and Technology Co., Ltd., of China is an exoskeleton robot that is kinematic equivalent to the human limb (Figure 1). In order to match natural redundancy and induce exact joint trajectories, the robot is with five degrees of freedom (DoF) to coincide with human upper extremity joints (shoulder and elbow). This feature is important to avoid mismatch undesired reaction forces that will be felt by participants as resistance to motion. There is one passive joint in the horizontal plane, while two active joints in the sagittal plane. For the passive joint in the horizontal plane, only active movement could be done by subjects. However, for the active joints of shoulder and elbow, either assistive or passive training could be provided. Active movement is done in degravity, while assisted or passive movement against gravity, which is conventional treatment by therapists for stroke survivors.

During rehabilitation training, the active joints consisting of motor and gear provide additional assistance to participants, with the encoder measuring joint angular information and the torque sensor obtaining human-robot interactive torque. We can develop different motion control algorithms to adapt different training modes based on the mechanical structure.

According to training requirement of upper extremity rehabilitation robot, the power unit on the sagittal plane of the joint is made up by a Maxon EC motor and a Harmonic Drive harmonic gear, which can provide additional 46 Nm and 13 Nm assistive torque to the shoulder and elbow joints, respectively. During training, the activity information of each degree of freedom (such as angle and angular velocity) can be measured corresponding to the encoder to control the active joint or saved in memory. The human-robot interactive force obtained by the torque sensor can be used to identify the movement intention of patient, which can improve the control precision of robot.

Our upper extremity robot has the ability to enable a strict application of motor learning principles known as stroke rehabilitation paradigms [13–17], which makes functional restoration by promoting neural plasticity and reorganization [18, 19]. Meanwhile, it makes provision of real-time sensory feedback and quantitative feedback for the participant correcting her/his movement and which is indispensable for impairment restoration [20]. Furthermore, center-out reaching of peripheral targets aimed at improving the coordination between shoulder and elbow achieves the goal-directed movement [21] during highly intensive training. The novel concept of closed-loop rehabilitation model of “central-peripheral-central” [22] is embodied during the robot-assisted rehabilitation process. Therefore, the rigorous application of motor learning principles, the goal-directed game, and the feedbacks described above make a promotion of active participation by stroke survivors, even in the passive teaching training for severely impaired [23].

### 2.1. Dynamic Model Analysis of Human-Robot System

**Figure 2:**
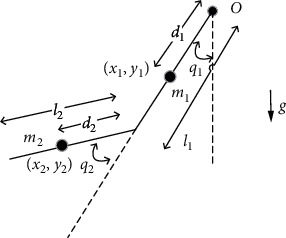
Simplified model of the human-robot system.

Figure 2 shows the simplified human-robot coupling dynamical model in the polar coordinate system. *g* stands for the acceleration of gravity; *m*_1_ represents the mass of shoulder module of robot and the shoulder of the patient. *d*_1_ is the length from the shoulder rotation center to center of mass, *l*_1_ is the length of upper arm of robot, and *q*_1_ is the shoulder angle. In the same way, *m*_2_, *d*_2_, *l*_2_, and *q*_2_ represent the same values of the elbow joint.

According to the Lagrange method, the above dynamic model could be analyzed and presented in mathematical expression as the following equation:

(1) $$\begin{array}{c} \tau_{1}=\left[m_{1}d_{1}^{2}+m_{2}\left(L_{1}^{2}+d_{2}^{2}\right)+2m_{2}L_{1}d_{2}\cos q_{2}\right]{\ddot{q}}_{1}+\left(m_{2}d_{2}^{2}+m_{2}L_{1}d_{2}\cos q_{2}\right){\ddot{q}}_{2}-2m_{2}L_{1}d_{2}\sin q_{2}\cdot {\dot{q}}_{1}{\dot{q}}_{2}-m_{2}L_{1}d_{2}\sin q_{2}\cdot {\dot{q}}_{2}^{2}+\left({\text{m}}_{1}{\text{d}}_{1}+{\text{m}}_{2}L_{1}\right)\text{g}\sin q_{1}+{\text{m}}_{2}\text{g}d_{2}\sin\left(q_{1}+q_{2}\right)\tau_{2}=\left(m_{2}d_{2}^{2}+m_{2}L_{1}d_{2}\cos q_{2}\right){\ddot{q}}_{1}+m_{2}d_{2}^{2}{\ddot{q}}_{2}+m_{2}L_{1}d_{2}\sin q_{2}\cdot {\dot{q}}_{1}^{2}+{\text{m}}_{2}\text{g}d_{2}\sin\left(q_{1}+q_{2}\right). \end{array}$$

*τ*_1_ and *τ*_2_ represent the driving moment of the shoulder and elbow, respectively.

### 2.2. Torque Controller

The torque control based on the position control is the global scheme of a robot controller. The robot completes the tracking of desired trajectory by introducing the feedforward controller and enhancing with a proportional differential controller. The Lagrange method-based human-robot coupling dynamical model in equation (1) is rewritten as

(2) $$\begin{array}{c} M\left(q\right)\ddot{q}+C\left(q,\dot{q}\right)+B\dot{q}+D\left(\dot{q}\right)+\text{G}\left(q\right)=\tau =\tau_{\text{m}}-\tau_{\text{h}}, \end{array}$$

where *τ* = [*τ*_1_, *τ*_2_] is the joint torque vector, *q* = [*q*_1_, *q*_2_] is the joint angular position vector, *M*(*q*) is the inertia matrix, $C\left(q,\dot{q}\right)$ is the Coriolis force matrix, G(*q*) is the grabity vector, *B* is the viscous friction vector, and *D* is the dynamic friction vector. *τ*_m_ is the torque generated by the motor; *τ*_h_ is the interaction torque vector.

**Figure 3:**
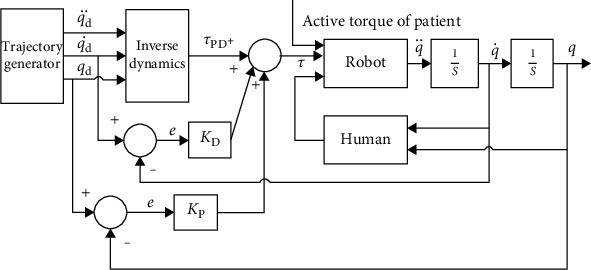
Structure of proportional differential-based trajectory tracking controller.

The scheme of proportional differential based on trajectory tracking controller is shown in Figure 3. According to the control scheme, we propose the control law as

(3) $$\begin{array}{c} \tau =\tau_{\text{PD}}\left({\ddot{q}}_{\text{d}},{\dot{q}}_{\text{d}},q_{\text{d}}\right)+K_{\text{D}}\dot{e}+K_{\text{P}}e, \end{array}$$

where *e*(*t*) = *q*(*t*) − *q*_d_(*t*), $\dot{e}\left(t\right)=\dot{q}\left(t\right)-{\dot{q}}_{\text{d}}\left(t\right)$, and *K*_P_ and *K*_D_ are proportional and differential coefficients of the proportional differential controller; the inverse dynamic term, $\tau_{\text{PD}}\left({\ddot{q}}_{\text{d}},{\dot{q}}_{\text{d}},q_{\text{d}}\right)$, is calculated by equation ((1)) and is the theoretical torque only. Since the torque control could enhance the smoothness significantly, it is essential to discuss the actual robot system. However, the actual robot system is determined by the frictions *B* and *D* in equation (2), which achieve the effects of torque control.

### 2.3. Friction Compensation

**Figure 4:**
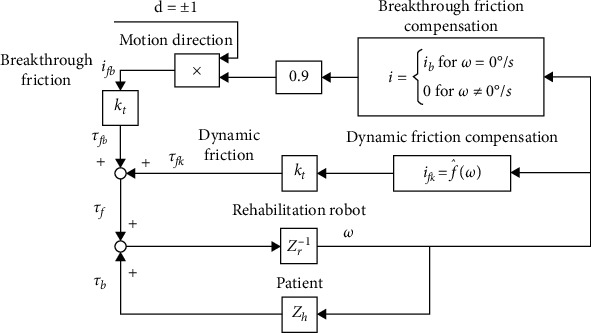
Schematic diagram of friction compensation.

The friction compensation principle is shown in Figure 4. For the breakthrough friction *i*_*f*_*b*__, compensation has occurred before the joint moves, while it cancels when the motion velocity of joint reduces to zero. *d* as the direction of breakthrough friction compensation is determined by the motion trend which is detected by torque sensors. The factor is set 0.9 to prevent the self-starting of the robot joint. Between the joint velocity *ω* and the dynamic friction torque *τ*_*f*_*k*__, a mathematical model $\hat{f}\left(\omega \right)$ is necessary to be established. The model is illustrated by the following equation according to a friction modeling method [24]. The constant coefficients are *c*_1_ ⋯ *c*_6_. For balancing the dynamic friction, the controller of robot identifies the parameters.

(4) $$\begin{array}{c} \tau_{f_{k}}=\hat{f}\left(\omega \right)=c_{6}\cdot \omega +\frac{c_{1}+e^{c_{2}\omega }-c_{3}}{c_{4}\cdot e^{c_{2}\omega }+c_{5}}. \end{array}$$

We calculate the frictions and gravity of robot under different positions and velocities by the proposed methods, balancing those values and improving the accuracy of the controller.

## 3. Teaching Training Mechanism

**Figure 5:**
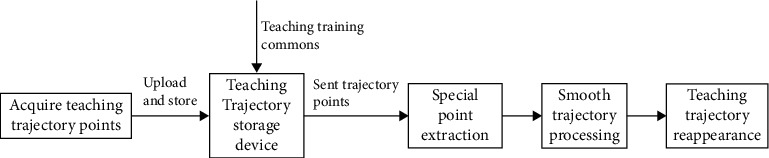
Teaching training control process.

Neurorehabilitation, as a clinical discipline, has been established to restore upper extremity motor function poststroke basing on the ability of training and physical activity [25]. At the early stage of recovery, even for patients with severe impairment, passive rehabilitation is strongly addressed. However, motor incoordination between the shoulder and elbow appears in the upper extremity rehabilitation robot currently. And movement trajectory is intermittent and deviates from the physiological activity of human due to application of general industrial methods. Furthermore, the motor is controlled by position merely and the upper extremity of a patient follows the robot arm strictly, which contribute to inflexibility. In this research, teaching training mechanism is developed to conquer the weaknesses, which includes three parts (Figure 5), namely, special point extraction, smooth trajectory processing, and teaching trajectory reappearance. Therefore, flexible and smooth movement facilitates by combining motion control with torque control.

### 3.1. Special Point Extraction

**Figure 6:**
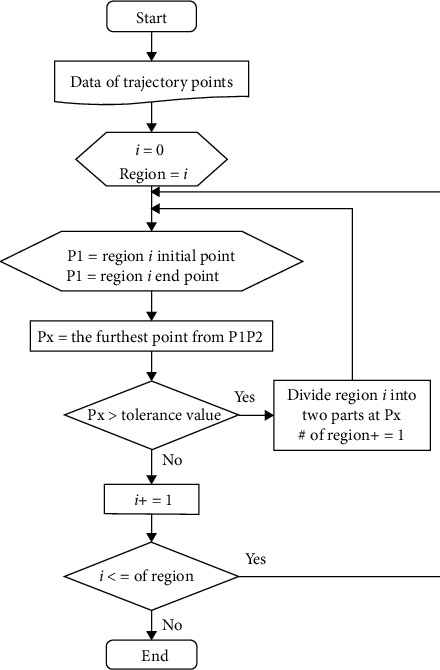
Special point extraction during teaching training.

The algorithm in Figure 6 is used to calculate the entered trajectory points during the teaching process. And then, special points of the trajectory will be output, which is processed into a smooth trajectory.

### 3.2. Smooth Trajectory Processing

Referring to the trajectory smoothing method and smoothing motion theory [26], the minimum joint acceleration is used in the smooth trajectory processing, with smooth and continuous variance of position, velocity, and acceleration. The trajectory function can be described by a fifth-power polynomial function in time as

(5) $$\begin{array}{c} x\left(t\right)=a_{0}+a_{1}t+a_{2}t^{2}+a_{3}t^{3}+a_{4}t^{4}+a_{5}t^{5}. \end{array}$$

*a*_0_ ⋯ *a*_5_ are constant coefficients of polynomial function powers. The first and second derivatives of the trajectory function with respect to time are

(6) $$\begin{array}{c} \dot{x}\left(t\right)=a_{1}+2a_{2}t+3a_{3}t^{2}+4a_{4}t^{3}+5a_{5}t^{4},\\ \ddot{x}\left(t\right)=2a_{2}+6a_{3}t+12a_{4}t^{2}+20a_{5}t^{3}. \end{array}$$

According to the boundary conditions, calculate the function value of $x\left(t_{0}\right),\dot{x}\left(t_{0}\right),\ddot{x}\left(t_{0}\right),x\left(t_{f}\right),\dot{x}\left(t_{f}\right),\ddot{x}\left(t_{f}\right)$ at the boundary. *t*_*i*_ and *t*_*f*_ are at the time of beginning and ending of the trajectory. When time is normalized, the function is expressed as

(7) $$\begin{array}{c} \tau =\frac{t_{f}-t_{0}}{D}. \end{array}$$

*τ* ∈ [0, 1], where *t* is the present moment, *t*_0_ is the initial time, *t*_*f*_ is the final time, and *D* = *t*_*f*_ − *t*_0_ and *dτ*/*dt* = 1/*D*. The trajectory function could be rewritten as

(8) $$\begin{array}{c} x\left(t\right)=a_{0}+a_{1}\tau +a_{2}\tau^{2}+a_{3}\tau^{3}+a_{4}\tau^{4}+a_{5}\tau^{5},\\ \dot{x}\left(t\right)=\frac{a_{1}}{D}+\frac{2a_{2}}{D}\tau +\frac{3a_{3}}{D}\tau^{2}+\frac{4a_{4}}{D}\tau^{3}+\frac{5a_{5}}{D}\tau^{4},\\ \ddot{x}\left(t\right)=\frac{2a_{2}}{D^{2}}+\frac{6a_{3}}{D^{2}}\tau +\frac{12a_{4}}{D^{2}}\tau^{2}+\frac{20a_{5}}{D^{2}}\tau^{3}. \end{array}$$

The initialization condition is $x\left(t_{0}\right)=x_{0}\;\dot{x}\left(t_{0}\right)=v_{0}\;\ddot{x}\left(t_{0}\right)=p_{0}$. When *t* = *t*_0_ and *τ* = 0, *a*_0_ = *x*_0_ *a*_1_ = *Dv*_0_ *a*_2_ = *D*^2^*p*_0_/2. The terminal constraint condition of movement is $x\left(t_{f}\right)=x_{f}\;\dot{x}\left(t_{f}\right)=v_{f}\;\ddot{x}\left(t_{f}\right)=p_{f}$. When *t* = *t*_*f*_ and *τ* = 1,

(9) $$\begin{array}{c} a_{3}=-\frac{3D^{2}}{2}p_{i}-6{Dv}_{i}+10\left(x_{f}-x_{i}\right)-4{Dv}_{f}+\frac{1}{2}D^{2}p_{f},\\ a_{4}=\frac{3D^{2}}{2}p_{i}+8{Dv}_{i}-15\left(x_{f}-x_{i}\right)+7{Dv}_{f}-D^{2}p_{f},\\ a_{5}=-\frac{D^{2}}{2}p_{i}-3{Dv}_{i}+6\left(x_{f}-x_{i}\right)-3{Dv}_{f}+\frac{1}{2}D^{2}p_{f}. \end{array}$$

The point-to-point continuous and smooth trajectory will be obtained by the above calculation, including position function, velocity function, and acceleration function. The teaching training is optimized by integrating the three functions. For the upper extremity robot, the above method is applied to extract the special points of the shoulder and elbow synchronously, basing on the dynamic model analysis of the human-robot system. Therefore, the shoulder and elbow trajectories of robot are synchronous at any time. The clinical application of the human-robot system works in the kinematics trajectory, imitating the voluntary movement of the human body.

### 3.3. Teaching Trajectory Reappearance

The global scheme of the upper extremity robot controller is based on the torque control by introducing the feedforward controller and enhancing with the proportional differential controller. The concrete algorithm has been described above.

During clinical rehabilitation, therapists give teaching trajectory by driving the impaired upper extremity of stroke survivors, which is based on residential function and joint motion patterns expected to enhance. Then, trajectory is conducted by adjusting the position, velocity, and acceleration, tending to smoothing and consecutiveness. Therefore, the coordination teaching trajectory will reappear repeatedly with high intensity. However, the travel velocity, training duration, and intensity are changed depending on the condition of patients, which achieves patient-centered customized training by the robot.

## 4. Experiments and Results

### 4.1. Experiment Scheme

In order to verify the effectiveness and efficacy of the proposed teaching training mechanism, functional and clinical experiments were schemed. A healthy volunteer was guided to carry out the functional experiments. In functional experiments, the subject was asked to keep the arm slack when testing the teaching trajectory, feigning upper extremity weakness. Moreover, a total of five stroke survivors were recruited to undergo the clinical experiments for investigating safety and efficacy. The experimental protocol was approved by the Ethics Committee of Jing'an District Centre Hospital of Shanghai, China.

### 4.2. Functional Experiments

The aim of function experiments is to test the designed control system with teaching training mechanism whether providing comfortable and flowing trajectory acquired from therapists. The subject is asked to keep the tested arm slack. As described above, trajectory is determined by therapists basing on the given origin and destination. Thus, the regulation integrated the dynamic model analysis of human-robot with functions of position, velocity, and acceleration. During the experiments, the corresponding information was recorded to verify the designed functions, such as the curve of trajectory, the range of shoulder or elbow, the velocity of moving, and the experience of the subject.

**Figure 7:**
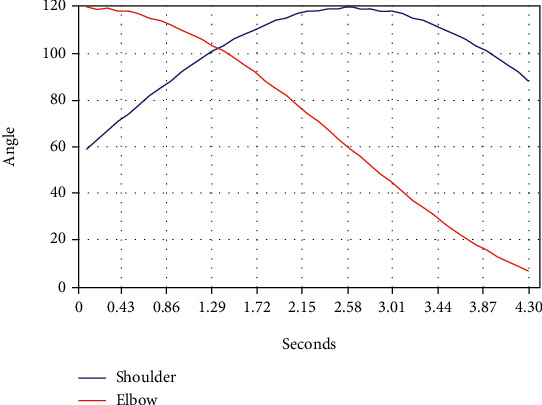
Teaching training trajectory. Shoulder flexion from 60 degrees in the sagittal plane to 120 degrees at 21 degrees per second along the axis of the coronal plane; Elbow flexion from 120 degrees to 8 degrees at 26 degrees per second.

Figure 7 shows the trajectory. The tested upper extremity moved from shoulder flexion 60 degrees in the sagittal plane to 120 degrees at 21 degrees per second along the axis of the coronal plane, with elbow flexion 120 degrees to 8 degrees at 26 degrees per second. Hence, the teaching training of shoulder flexion with elbow extension presents the Bobath approach [27]

The approach is a classic theory for hemiplegia rehabilitation after stroke and is worthy of extensive clinical application by therapists. Additionally, no pain and discomfort occurred in the subject during the test. We tentatively suggest that this designed control strategy with the teaching training mechanism could provide effective and safe training.

### 4.3. Clinical Experiments

The aim of clinical experiments is to verify the effects of the proposed teaching training. Clinical experiments with five recruited stroke survivors are conducted with teaching training customized on the residual functional ability of stroke survivors during robot-assisted therapy last for four weeks (more than 3 times per week and 15 training days totally). Each survivor undergoes one session in passive training of the shoulder and elbow, 30 min per session, one training day. The program also included two-hour daily (5 days a week) sessions of physical and occupational therapy based on the paretic extremity rehabilitation and, if necessary, half an hour of speech therapy 5 times a week. Motor impairment was measured using the active range of motion (AROM) of the shoulder and elbow and the upper limb Fugl-Meyer [28] Assessment for the shoulder/elbow and coordination (Fugl-Meyer SEC) was performed before training, at midterm (after the fifth training session), and after the last session. The AROM as the sum of shoulder and elbow movements (shoulder flexion/extension, adduction/abduction, and elbow flexion/extension) was used to assess the joint excursion which could be considered correlated to spasticity [29].

The Fugl-Meyer scale measured the ability to move paretic arm, which is a global evaluation scale for impairment in stroke patients. Fugl-Meyer SEC includes items related to the movements of shoulder and elbow and the coordination. Each item is rated on a 3-point scale (maximum score, 42 points).

Demographics and clinical characteristics of five stroke survivors are presented in Table 1, with four males and one female. Subjects were between 1 month and half a year poststroke at the time they enrolled in the study with two affected on their dominant side, while three with cerebral hemorrhage and two with cerebral infarction; the Fugl-Meyer SEC of subjects ranged from 9 to 27. All subjects exhibited moderate-to-severe deficits in movement capabilities of the paretic extremity. No other current significant impairment of the upper extremity exists in all stroke survivors, e.g., fixed contracture, frozen shoulder, severe arthritis, recent fracture, or bleeding.

**Figure 8:**
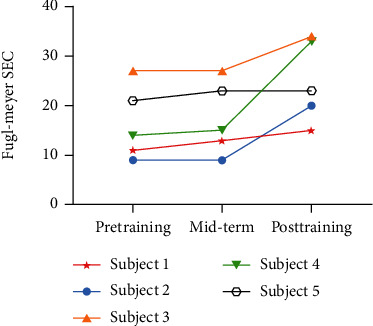
Compared Fugl-Meyer SEC of survivors. Fugl-Meyer SEC: Fugl-Meyer assessment for shoulder/elbow and coordination; Mid-term: after the fifth training session.

All survivors regained an increased Fugl-Meyer SEC score after teaching training. Subject 1 improved in flexor and extensor synergy of the upper extremity. Subjects 2 and 4 improved in flexor and extensor synergy, movement combing, and out of synergy. Subject 3 was without improvement in extensor synergy for the ceiling effect. Subject 5 remained powerless in movement combining and out of synergy (Table 2), while subject 2 and subject 4 increased more obviously after midterm. No improvement appeared for subjects 2 and 3 before midterm. However, subject 5 reached a plateau after midterm and with the least progress (Figure 8).

**Figure 9:**
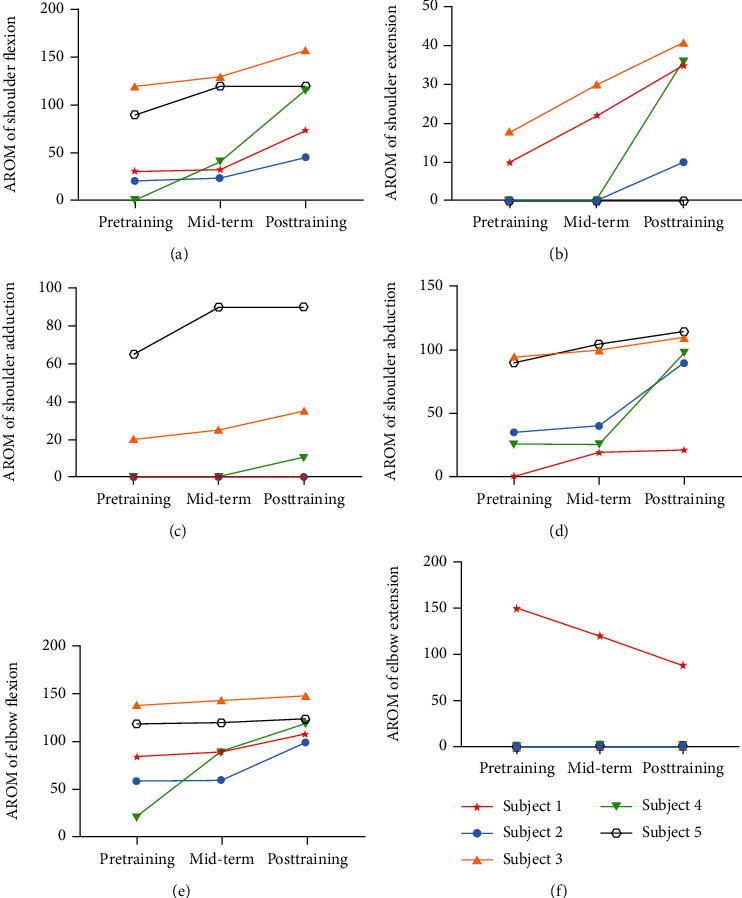
Compared AROM of the shoulder and elbow. AROM: active range of motion; Mid-term: after the fifth training session.

The AROM of the shoulder (flexion and extension, adduction, and abduction) and elbow (flexion and extension) are displayed in Figure 9. The five stroke survivors improved AROM of flexion for the shoulder and elbow and abduction for the shoulder. However, one subject in the AROM of shoulder extension, two subjects in shoulder adduction, and four subjects in elbow extension appeared to have no improvement. Before midterm, no restoration of shoulder AROM presented for subjects 1 and 2 in flexion, subjects 2 and 5 in extension, and subjects 1, 2, and 4 in adduction. The similar situation occurred in elbow flexion for subjects 2 and 5 and extension for subjects 2, 3, 4, and 5. Nevertheless, the AROM change of shoulder extension in subject 5 and shoulder adduction in subject 1 was vacant. The variation in the AROM of elbow extension was unique during training, with only subject 1 benefitting from the teaching training and other subjects maintaining. Despite the fact that the effectiveness is not for all subjects, no adverse events and unsatisfactory events occurred during robot-assisted teaching training. No trajectory against kinematics of the human body appeared during experiments.

## 5. Conclusion and Discussion

The artificial intelligence and the technological revolution seem to indicate a greater artificial cognitive agents in our clinical practice, especially in rehabilitation poststroke. Robot-assisted therapy for upper extremity rehabilitation with effective scientific protocol achieves motor function recovery. Passive training with the upper extremity robot is crucial for stroke survivors with greater impairment of the upper extremity who have difficulty moving actively and even at the early stage. In the investigation, a control strategy with the teaching training mechanism was proposed to realize customized passive neurorehabilitation exercise, serving with safety and efficacy robot-assisted motion. The teaching training was developed to assist therapists by supplying customized passive training, ensuring coordination and smoothness of upper extremity motor. Meanwhile, the robot-assisted teaching training offers passive training sufficiently and alleviates the shortage of therapists.

The teaching trajectory motion keeps shoulder and elbow movements synchronized and coordination during the course of repeated movements, resolved with position and torque controlling simultaneously. Meanwhile, the model of the human-robot system is adopted to extract special points, avoiding the violation of upper extremity normal movement. Furthermore, the subject could resist the detailed movement by arm strength to realize the flexibility and plasticity of movement, with no need to follow the trajectory strictly.

The functional experiments and clinical experiments were schemed and investigated with a healthy adult and five recruited stroke survivors, respectively. In the functional experiment, the repetitive passive training effectively inhibits the formation of joint adhesions, inducing the impairment of range of joint movement. In addition, the teaching training showed a smooth and coordinating curve based on the Bobath approach. The trajectory is used to suppress abnormal pattern by correcting the upper extremity flexor spasticity, while improving joint mobility and activities of daily living. During clinical experiments, the upper extremity function was evaluated by the Fugl-Meyer SEC and AROM of the shoulder and elbow. The improvement of upper extremity functional evaluation by the Fugl-Meyer SEC unfolds the restoration of the upper extremity function by robot-assisted teaching training. However, not all subjects achieve remission on the AROM of the shoulder and elbow, especially in extension. The outcomes are coinciding with the impairment of stroke. The experimental results showed that the suggested control strategy not only serves with safe teaching training but also presents rehabilitation efficacy. Due to the small sample size, the result should be cautiously considered.

Therefore, the developed teaching training mechanism played an important role to serve subjects with customized training, as closely as what the therapist did. The trajectory is made by therapists based on the residual function, ensuring adherence to the principle of human kinematics. Future models should be updated according to the clinical demands. Beyond the shoulder and elbow joints, the wrist and fingers are a nonnegligible fraction of the upper extremity function. A robot integrating shoulder, elbow, wrist, and fingers with human anatomy mechanics is indispensable in clinical rehabilitation. So it is worth investigating in future studies. On top of this, the customization of active and assistive models during robot-assisted training is equally important, which makes the robot an ideal therapist. And further study will investigate the rehabilitation efficacy with controlled experiments.

## Figures and Tables

**Table 1:** Information of stroke survivors.

Patient code	Age	Gender	Type of stroke	Days since stroke	Impaired extremity	MMSE	Fugl-Meyer SEC
S1	37	Male	CH	162	Left	30	11
S2	66	Female	CH	46	Left	24	9
S3	30	Male	CH	178	Right	29	27
S4	43	Male	CI	26	Left	26	14
S5	57	Male	CI	29	Right	25	21

CH: cerebral hemorrhage; CI: cerebral infarction; MMSE [30]: mini-mental state examination; Fugl-Meyer SEC: Fugl-Meyer assessment for shoulder, elbow, and cooperation.

**Table 2:** Subitem scores of Fugl-Meyer SEC.

Patient code	Reflex activity		Flexor synergy		Extensor synergy		Movement combining synergies		Movement out of synergy		Normal reflexes (sitting)		Coordination	
	Pre	Post	Pre	Post	Pre	Post	Pre	Post	Pre	Post	Pre	Post	Pre	Post
S1	4	4	3	6	0	1	0	0	0	0	0	0	4	4
S2	4	4	2	6	1	3	0	1	0	2	0	0	2	4
S3	4	4	10	11	6	6	3	5	2	4	0	0	2	4
S4	4	4	3	12	3	5	0	5	0	3	0	0	4	4
S5	4	4	10	11	3	4	0	0	0	0	0	0	4	4

Fugl-Meyer SEC: Fugl-Meyer assessment for shoulder/elbow and coordination; Pre: pretraining; Post: posttraining.

## Data Availability

The data that support the findings of the study are available from the corresponding author on reasonable request.

## References

[1] Morone G., Cocchi I., Paolucci S., Iosa M. (2020). Robot-assisted therapy for arm recovery for stroke patients: state of the art and clinical implication. *Expert Review of Medical Devices*.

[2] Mehrholz J., Pohl M., Platz T., Kugler J., Elsner B. (2015). Electromechanical and robot-assisted arm training for improving activities of daily living, arm function, and arm muscle strength after stroke. *Cochrane Database of Systematic Reviews*.

[3] Klamroth-Marganska V. (2018). Stroke Rehabilitation: Therapy Robots and Assistive Devices. *Sex-Specific Analysis of Cardiovascular Function*.

[4] Langhorne P., Bernhardt J., Kwakkel G. (2011). Stroke rehabilitation. *Lancet*.

[5] Prange G. B., Jannink M. J. A., Groothuis-Oudshoorn C. G. M., Hermens H. J., IJzerman M. J. (2006). Systematic review of the effect of robot-aided therapy on recovery of the hemiparetic arm after stroke. *Journal of Rehabilitation Research and Development*.

[6] Lemon R. N., Porter R. (1976). Afferent input to movement-related precentral neurones in conscious monkeys. *Proceedings of the Royal Society of London - Series B: Biological Sciences*.

[7] Naito E., Roland P. E., Ehrsson H. H. (2002). I feel my hand moving: a new role of the primary motor cortex in somatic perception of limb movement. *Neuron*.

[8] Dechaumont-Palacin S., Marque P., De Boissezon X. (2008). Neural correlates of proprioceptive integration in the contralesional hemisphere of very impaired patients shortly after a subcortical stroke: an FMRI study. *Neurorehabilitation and Neural Repair*.

[9] Lindberg P., Schmitz C., Forssberg H., Engardt M., Borg J. (2004). Effects of passive-active movement training on upper limb motor function and cortical activation in chronic patients with stroke: a pilot study. *Journal of Rehabilitation Medicine*.

[10] Lewis G. N., Byblow W. D. (2004). The effects of repetitive proprioceptive stimulation on corticomotor representation in intact and hemiplegic individuals. *Clinical Neurophysiology*.

[11] Carel C., Loubinoux I., Boulanouar K. (2000). Neural substrate for the effects of passive training on sensorimotor cortical representation: a study with functional magnetic resonance imaging in healthy subjects. *Journal of Cerebral Blood Flow and Metabolism*.

[12] Weiller C., Jüptner M., Fellows S. (1996). Brain representation of active and passive movements. *NeuroImage*.

[13] Kwakkel G., Kollen B. J., Krebs H. I. (2008). Effects of robot-assisted therapy on upper limb recovery after stroke: a systematic review. *Neurorehabilitation and Neural Repair*.

[14] Kitago T., Krakauer J. W. (2013). Motor learning principles for neurorehabilitation. *Handbook of Clinical Neurology*.

[15] Huang V. S., Krakauer J. W. (2009). Robotic neurorehabilitation: a computational motor learning perspective. *Journal of Neuroengineering and Rehabilitation*.

[16] Krakauer J. W. (2006). Motor learning: its relevance to stroke recovery and neurorehabilitation. *Current Opinion in Neurology*.

[17] Piron L., Turolla A., Agostini M. (2010). Motor learning principles for rehabilitation: a pilot randomized controlled study in poststroke patients. *Neurorehabilitation and Neural Repair*.

[18] Sampaio-Baptista C., Sanders Z. B., Johansen-Berg H. (2018). Structural plasticity in adulthood with motor learning and stroke rehabilitation. *Annual Review of Neuroscience*.

[19] Nudo R. J. (2003). Adaptive plasticity in motor cortex: implications for rehabilitation after brain injury. *Journal of Rehabilitation Medicine*.

[20] Subramanian S. K., Massie C. L., Malcolm M. P., Levin M. F. (2010). Does provision of extrinsic feedback result in improved motor learning in the upper limb poststroke? A systematic review of the evidence. *Neurorehabilitation and Neural Repair*.

[21] Winstein C., Lewthwaite R., Blanton S. R., Wolf L. B., Wishart L. (2014). Infusing motor learning research into neurorehabilitation practice: a historical perspective with case exemplar from the accelerated skill acquisition program. *Journal of Neurologic Physical Therapy*.

[22] Jia J. (2016). Closed-loop rehabilitation model of "central-peripheral-central"-novel concept of hand rehabilitation after stroke. *Chinese rehabilitation medical journal*.

[23] Cano-de-la-Cuerda R., Molero-Sánchez A., Carratalá-Tejada M. (2015). Theories and control models and motor learning: clinical applications in neuro-rehabilitation. *Neurología*.

[24] Nef T., Lum P. (2009). Improving backdrivability in geared rehabilitation robots. *Medical & Biological Engineering & Computing*.

[25] Nudo R. J., Wise B. M., SiFuentes F., Milliken G. W. (1996). Neural substrates for the effects of rehabilitative training on motor recovery after ischemic infarct. *Science*.

[26] Flash T., Hogan N. (1985). The coordination of arm movements: an experimentally confirmed mathematical model. *Journal of Neuroscience*.

[27] Burton C. V., Lozano H. D., Werssowetz O. F. V. O. N., Zedler E. Y. (1956). Experiences with Bobath method of treatment of cerebral palsy. *Archives of Physical Medicine and Rehabilitation*.

[28] Fugl-Meyer A. R., Jääskö L., Leyman I., Olsson S., Steglind S. (1975). The post-stroke hemiplegic patient. 1. A method for evaluation of physical performance. *Scandinavian journal of rehabilitation medicine*.

[29] Sommerfeld D. K., Gripenstedt U., Welmer A. K. (2012). Spasticity after stroke: an overview of prevalence, test instruments, and treatments. *American Journal of Physical Medicine & Rehabilitation*.

[30] Galasko D., Klauber M. R., Hofstetter C. R., Salmon D. P., Lasker B., Thal L. J. (1990). The mini-mental state examination in the early diagnosis of Alzheimer's disease. *Archives of Neurology*.

